# The Pathophysiology of Inherited Renal Cystic Diseases

**DOI:** 10.3390/genes15010091

**Published:** 2024-01-11

**Authors:** Matthew Satariano, Shaarav Ghose, Rupesh Raina

**Affiliations:** 1 Department of Medicine, Northeast Ohio Medical University, Rootstown, OH 44272, USA; msatariano@neomed.edu (M.S.); sghose@neomed.edu (S.G.); 2 Akron Nephrology Associates, Cleveland Clinic Akron General Medical Center, Akron, OH 44307, USA; 3 Department of Nephrology, Akron Children’s Hospital, Akron, OH 44308, USA

**Keywords:** interstitial diseases, cystic diseases, renal, kidney, autosomal dominant polycystic kidney disease, autosomal recessive polycystic kidney disease, autosomal dominant tubulointerstitial kidney disease, multicystic dysplastic kidney disease, cystic dysplasia, Zellweger syndrome, calyceal diverticula, nephronophthisis, Bardet–Biedl syndrome, Meckel–Gruber syndrome

## Abstract

Renal cystic diseases (RCDs) can arise from utero to early adulthood and present with a variety of symptoms including renal, hepatic, and cardiovascular manifestations. It is well known that common RCDs such as autosomal polycystic kidney disease and autosomal recessive kidney disease are linked to genes such as PKD1 and PKHD1, respectively. However, it is important to investigate the genetic pathophysiology of how these gene mutations lead to clinical symptoms and include some of the less-studied RCDs, such as autosomal dominant tubulointerstitial kidney disease, multicystic dysplastic kidney, Zellweger syndrome, calyceal diverticula, and more. We plan to take a thorough look into the genetic involvement and clinical sequalae of a number of RCDs with the goal of helping to guide diagnosis, counseling, and treatment.

## 1. Introduction

Renal cystic diseases (RCDs), with an incidence ranging from 0.44–4.1 per 10,000 births, can impact individuals of all age groups and lead to severe complications such as chronic kidney disease (CKD), liver disease, and death [1]. The classification of RCDs varies based on factors such as inheritance pattern (genetic vs. sporadic) and ciliopathies or dysplasias. While the genetic associations of common RCDs like autosomal dominant polycystic kidney disease (ADPKD) and autosomal recessive polycystic kidney disease (ARPKD) are well established, the genetic pathophysiology of less-studied RCDs must be explored. We plan to comprehensively examine the genetic involvement and clinical outcomes of various RCDs to help contribute valuable insights for diagnosis, counseling, and treatment. 

## 2. Renal Embryology

The kidney originates from the intermediate mesoderm in a three-stage process: the pronephros, mesonephros, and metanephros. First, the formation of the pronephros begins in the third week of gestation, in which the nephrotomes merge to create a pronephric duct. Subsequently, the mesonephros develops caudally in relation to the pronephros. The emergence of the pronephric duct initiates the development of mesonephric tubules which drain into the mesonephric duct [2]. Regression of the pronephric duct occurs by the conclusion of the fourth week of gestation. Simultaneously, the mesonephric duct induces the formation of the ureteric bud, which plays a pivotal role in the development of the mature kidney. At the end of the second month, the mesonephric duct will regress. The mature kidney evolves from the metanephros, a process commencing in the fifth week and reaching functionality by the twelfth week [2]. The metanephric system is comprised of structures including the excretory and collecting system. The excretory system contains components such as the glomerulus, Bowman’s capsule, proximal and distal convoluted tubules, loop of Henle, and segments of the collecting tubule. The collecting system originates from the ureteric bud and comprises the ureter, kidney pelvis, minor and major calyces, and the collecting tubules [2]. The production of urine occurs by 16 weeks of gestation and the fetal bladder is visible by week 9 of gestation Fetal kidneys can become detectable with ultrasound by 10–12 weeks of gestation, displaying a decreasing echogenicity throughout fetal growth, which results in corticomedullary differentiation at birth [2].

When analyzing renal abnormalities, ultrasonography allows for a comprehensive analysis of all aspects of renal appearance including the presence or absence of kidneys, their size and location, and their echogenicity. Kidney ultrasonography scans are commonly assessed in two planes: sagittal for growth evaluation, and transverse for examination of the renal pelvis. Starting from 13 weeks, bladder imaging can be conducted if the patient maintains a full bladder [1].

## 3. Genetic Testing and Patient Counseling

### 3.1. Genetic Testing and Possible Outcomes

Genetic testing may be conducted using nontargeted and targeted next-generation sequencing (NGS). Enhancing our knowledge of the genetics behind kidney disorders can offer insights into the clinical management, prognosis, and likelihood of recurrence in affected individuals. In cases of severe conditions, such as the presence of anhydramnios and significant pulmonary underdevelopment in the fetus, the option of terminating the pregnancy may be considered when postnatal survival is deemed impossible [1].

### 3.2. Implications of Possible Results for Patient and Family

When a kidney anomaly is detected, prenatal counseling with geneticists, maternal-fetal medicine specialists, and neonatologists should be provided. The consultation should include exploring family history, discussing potential treatments and disease prognosis, and the recommendation of genetic testing [1]. Counseling remains crucial in less severe cases wherein children may require dialysis or other intensive care management at birth. Genetic testing and counseling are essential whenever prenatal ultrasonography reveals a renal anomaly, in order to help assemble an appropriate treatment plan [3].

### 3.3. Testing Other Family Members

It is important to consider the ethical, legal, and social implications (ELSI) when thinking about genetic testing and family members [4]. The clinician should ask themselves whether there is a clinical utility and added health or psychological benefit in testing family members. Some research suggests defining utility as health-related quality of life (QoL) [4]. In fact, these health economists suggest quantifying both QoL and survival into quality-adjusted life years (QALYs) when considering whether or not to test family members [4]. In terms of reproductive implications, some research suggests it is important to perform prenatal genetic testing for improving the overall outcomes of the mother and family [5]. There are more in utero therapies under trial that could be beneficial going forward for previously untreatable conditions. Thus, as more studies come out, genetic testing could play a larger role in reproductive ethics.

## 4. Dysplasias

### 4.1. Multicystic Dysplastic Kidney

#### 4.1.1. Genetics/Pathophysiology

Multicystic dysplastic kidney (MCDK) represents a type of kidney dysplasia characterized by the presence of both smaller and larger cysts from undifferentiated and immature tissue. MCDK stands out as a common cause of abdominal masses in neonates, impacting approximately 1 in every 1000 to 4000 live births [6]. MCDK is attributed to pathogenic variations in specific development genes, including *TCF2 and PAX2*, and mutations involving uroplakins [1]. MCDK involves a cystic form along with a hydronephrotic form that typically has an identifiable renal pelvis. The development of MCDK is associated with malformation of the ureteric bud branches and ampullae due to the presence of congenital ureteral obstruction during early nephrogenesis [7]. This may lead to significant atresia or complete disconnection between the ureteric bud and renal blastema, ultimately preventing the release of bud-branching signaling factors needed for proper nephron development [7]. Bilateral MCDK is frequently linked to a genetic origin, whereas unilateral MCDK occurs sporadically. One case report of MCDK in a family suggests the inherited form demonstrated an autosomal dominant inheritance pattern [8]. Pregnancies identified early with fetal bilateral MCDK are at higher risk, and may require frequent monitoring for conditions such as oligohydramnios and pulmonary hypoplasia [1].

#### 4.1.2. Presentation and Diagnosis 

Ultrasonographic imaging is the primary method for detecting MCDK, with bright kidneys being identified by antenatal ultrasonography 94% of the time [1]. MCDK can manifest unilaterally or bilaterally, with unilateral MCDK occurring more frequently than bilateral MCDK. Male fetuses more commonly exhibit unilateral MCDK [9]. Unilateral MCDK is associated with contralateral renal abnormalities in 7–43% of cases [10]. Neonates with MCDK may exhibit small kidneys due to underdeveloped glomeruli and primitive tubules. MCDK is linked to various diseases including Zellweger syndrome, renal–hepatic–pancreatic dysplasia, branchio-oto-renal syndrome, BBS, VACTERL, Eagle–Barret Syndrome, and renal coloboma syndrome [1].

High-resolution prenatal ultrasonography effectively enables early diagnosis of MCDK, while postnatal assessments involve diuretic renograms to evaluate renal function. Approximately 7% to 14% of pregnancies suspected to have MCDK exhibit chromosomal abnormalities and syndromes [11].Chromosome microarray analysis (CMA) is a diagnostic tool that identifies submicroscopic copy number variations (CNVs), which are deletions or repetitions in DNA that can cause different clinical presentations. Raina et al. reported CMA detection rates of 15% in isolated MCDK cases and 20% in those with extra renal symptoms, suggesting its potential to enhance detection of MCDK. In a study with kidney disease patients, pathogenic variants in 68% of patients were identified through whole-exome sequencing. This study demonstrates that whole-exome sequencing holds potential as an emerging diagnostic tool for inherited renal cystic diseases [12].

#### 4.1.3. Management

Neonates with MCDK require regular assessments of serum creatinine and blood pressure to detect potential CKD. In cases of unilateral MCDK, monitoring for appropriate contralateral kidney hypertrophy via ultrasonography is crucial at various intervals, including the neonatal stage and ages 2, 5, and 10. Furthermore, periodic urinalysis is recommended to identify proteinuria, and monitoring of vesicoureteral reflux is necessary to prevent damage to the contralateral kidney from urinary tract infections [1]. However, the majority of cases can resolve over time without necessitating surgical intervention [11].

### 4.2. Cystic Dysplasia 

#### 4.2.1. Genetics/Pathophysiology

Cystic dysplasia is defined by the presence of one or more cysts in the fetal kidney, diminished renal size, the absence of corticomedullary differentiation, and hyperechogenic parenchyma. Cystic dysplasia is considered to be rare, with an estimated incidence of 0.2–0.5% [13]. Although renal cystic dysplasia exhibits resemblances to MCDK, it is essential to distinguish the two disorders to ensure accurate diagnosis and proper management. Although the disorder frequently affects the entire kidney, some cases may manifest in only a singular renal segment. While cystic dysplasia can occur independently, it may be linked to genetic disorders such as Mullerian duct aplasia, renal coloboma syndrome (mutation in PAX2), heart defects, atresia choanae, renal aplasia, cervicothoracic somite dysplasia (MURCS), growth retardation, genital and ear abnormalities (CHARGE), and BOR syndrome (mutations in EYA1, SIX1, and SIX5) [1]. Additionally, urologic malformations such as Eagle–Barret syndrome and posterior urethral valve (PUV) disease may be linked to cystic dysplasia. 

#### 4.2.2. Presentation and Diagnosis

The presentation of cystic dysplasia is similar to MCDK, and differentiation via imaging and evaluation of the entire fetal body provides for optimal diagnostic accuracy [7]. Mercaptoacetyltriglycine (MAG3) scintigraphy can also be utilized to help distinguish dilated calyces and dysplastic cysts [1]. 

#### 4.2.3. Management 

The distinction between MCDK and cystic dysplasia is imperative for proper counseling and management. The management of cystic dysplasia revolves around regular monitoring of blood pressure and proteinuria, as these measures are indicative of disease progression and complications like irreversible hypertension and renal dysfunction [7].

### 4.3. Zellweger Syndrome

#### 4.3.1. Genetics/Pathophysiology

Zellweger syndrome is an autosomal recessive disorder characterized by mutations in peroxisomal biogenesis-related PEX genes. The incidence of Zellweger syndrome in the US is estimated to be 1 in 50,000 live births [14]. Approximately 80% of cases of Zellweger syndrome are linked to PEX1, PEX6, and PEX26, genes which contribute to the recycling system of peroxisomal target signaling (PTS) receptors. Meanwhile the translocation membrane system is encoded by PEX2, PEX10, and PEX12, genes which are mutated in about 10% of cases [15]. Peroxisomes play a crucial role in various cellular processes, including the breakdown of very-long-chain fatty acids (VLCFA) via β-oxidation and the breakdown of branched-chain fatty acids via α-oxidation. Additionally, they are involved in the catabolism of ethanol and amino acids, as well as the synthesis of steroid hormones and bile acids [14]. Additionally, peroxisomes contribute to the degradation of harmful hydrogen peroxide. When peroxisomes are dysfunctional, there is an accumulation of VLCFAs, reactive oxygen species, and bile acid intermediates, which can contribute to the pathogenesis of Zellweger syndrome [14].

#### 4.3.2. Clinical Presentation and Diagnosis

Typical manifestations of Zellweger syndrome include atypical facial and skeletal features, muscle tone loss, neocortical dysplasia, epileptic seizures, abnormal head size, and renal cysts in about 70% of cases [1]. Newborn screening is typically when the diagnosis of Zellweger syndrome is conducted through the recognition of clinical characteristics and elevated VLCFA levels. The diagnosis is confirmed through the genetic testing of PEX genes using whole-exome or whole-genome sequencing. Additionally, biochemical testing can examine increased levels of VLCFA, phytanic and/or pristanic acid, pipecolic acid, and other bile acid intermediates [14]. 

#### 4.3.3. Management

Treatment typically focuses on supportive care; however, Cholbam (cholic acid) was recently approved to treat Zellweger syndrome patients with liver manifestations [16].

### 4.4. Calyceal Diverticula

#### 4.4.1. Genetics/Pathophysiology

Calyceal diverticula (CD) are rare anomalies characterized by pouch-like protrusions of the calyx into the renal parenchyma, which increase the risk of nephrolithiasis and infection. CD has a similar prevalence in both adult and pediatric populations, estimated to be 0.21% to 0.6% of individuals undergoing intravenous urograms (IVU) [17]. The pathophysiology leading to CD development remains unclear, but potential causes include regression of ureteric buds due to inflammation, infection, or VUR-related obstruction [18]. The transitional epithelium of these diverticula enables the passive flow of urine to fill the neck of the diverticula. Acquired CD can develop secondary to obstructive, traumatic, neuromuscular, or fibrotic factors. While CD is not strictly classified as a genetic kidney cystic disease, it has been associated with mutations including EYA1 and SIX1, which are linked to conditions like BOR syndrome, or other causes of congenital anomalies of the kidney and urinary tract (CAKUT) [1]. The existing literature indicates that diverticula within the pelvicalyceal system and epidermoid cysts may manifest as congenital abnormalities arising in the embryonic phase [18].

#### 4.4.2. Clinical Presentation and Diagnosis

CD can be identified as early as 35 days into pregnancy and is divided into type I and type II. Type I is the more common form and is typically located in the upper pole of the kidney, and interacts with infundibulum or minor calyx. Type II is the larger and symptomatic form that is found in the center of the kidney, interacting with the major calyx or renal pelvis. Typically, CD stones are calcium oxalate, with a median stone size of 10 mm [19]. Although CD does not usually cause severe symptoms, it may lead to macrohematuria, UTIs, or flank pain. Ultrasonographic imaging and intravenous or computed tomography urography (CTU) are commonly employed for CD diagnosis [17]. A retrospective study found that the success of treatment was influenced by the timing of CD diagnosis [19].

#### 4.4.3. Management

Shockwave lithotripsy is employed for neonates with CD and nephrolithiasis, while smaller diverticula are addressed with minimally invasive endoscopic procedures, and larger ones are managed with laparoscopic procedures [17]. The management of CD varies based on the size and location of the diverticula [19].

## 5. Hepatorenal Fibrocystic Diseases (HRFCDs)

### 5.1. Autosomal Dominant Polycystic Kidney Disease

#### 5.1.1. Genetics/Pathophysiology

Autosomal dominant polycystic kidney disease (ADPKD) stands out as the most prevalent genetic disorder among adults, impacting an estimated 1 in 500–2500 [20]. ADPKD is primarily associated with the common loci *PKD1* and *PKD2*, which, respectively, account for approximately 78% and 15% of cases. Other genes such as *GANAB*, *DNAJB11*, *ALG9*, and *IFT140* contribute to a smaller percentage of cases [21]. PKD1 and PKD2 encode for polycystin-1 (PC1) and polycystin-2 (PC2), respectively [22]. PC1 is a 450-kD receptor-like protein characterized by a substantial extracellular N terminus, 11 membrane-spanning domains, and a brief cytoplasmic C terminus. The expression of PC1 is notably high in fetal renal tissue and relatively low in adult tissue. The N terminus of PC1 includes 15 PKD repeat motifs, a C-type lectin domain, and two leucine-rich motifs, collectively all playing a vital role in placing PC1 within the plasma membrane and junctional complexes. The C-terminal tails (CTT) allow PC1 and PC2 to form a complex allowing for the regulation of intracellular Ca^2+^ [22]. The CTT of PC1 consists of a G protein-binding domain, a coiled-coil domain, and components related to degradation. During the early secretory pathway, the N- and C-terminal domains can be cleaved. Specifically, the N-terminal is cleaved at the G protein-coupled receptor proteolysis site. Although PC1 exists in different lengths of cleaved forms, some studies propose that N-terminal cleavage is vital for its activation and function [22].

PC2, a six-transmembrane protein with intracellular N and C termini, functions as a Ca^2+^ responsive cation channel within the transient receptor potential family. The primary role of PC2 is to release calcium from the intracellular stores. The PC1 and PC2 complex becomes active, and initiates signal transduction when the cilium bends due to mechanical or chemical stimuli [22]. ADPKD may result from a missense mutation in the conducting pore located between the fifth and sixth transmembrane domains. PC2, inositol 1,4,5-triphosphate receptor (IP3R), and ryanodine receptors all serve as an indirect controller of cytoplasmic calcium levels. The C-terminus of PC2 interacts with IP3R, allowing for IP3-mediated calcium influx, while also binding to the ryanodine receptor channel to manage calcium-induced calcium release. PC2 is mainly found in the endoplasmic reticulum (ER) and early Golgi body within cells, but its specific location relies on the proteins binding to its C terminus. The movement of PC2 from the ER to the Golgi is influenced by a protein called polycystin-2 interactor (PIGEA-14) [23].

Several studies indicate that the specific locations of PC1 and PC2 are dependent on each other. This was demonstrated through a study confirming that impairing the function of PC1 prevented GPS cleavage in ADPKD cyst cells, ultimately leading to decreased amount of PC1 and PC2 in primary cilia. PC2 inhibits the activation of G proteins by PC1 through their physical connection mediated by their CTTs [24]. 

#### 5.1.2. Signaling Pathways

The development of ADPKD is linked to the malfunction of PC1 and/or PC2 proteins, which can affect various signaling pathways such as planar cell polarity (PCP), mammalian target of rapamycin (mTOR), G-protein coupled receptor (GPCR), epidermal growth factor receptor (EGFR), cystic fibrosis transmembrane conductance regulator (CFTR), Wnt, mitogen-activated protein kinase (MAPK), cellular Ca^2+^, and the cell cycle [22]. Due to the role PC1 and PC2 play in Ca^2+^ homeostasis, disturbances in function reduce intracellular Ca^2+^ levels, causing increased cyclic adenosine monophosphate (cAMP) signaling and cell proliferation. In polycystic kidney disease (PKD), elevated cAMP levels have not been localized to the kidney, and have been seen in other tissues [22]. Various hypotheses have been suggested to explain how cAMP levels are influenced in PKD, involving phosphodiesterase and adenylyl cyclases (ACs) [22].

The Wnt signaling pathways are categorized into canonical (β-catenin-dependent) and non-canonical (β-catenin-independent) pathways and function in cell growth, planar cell polarity, and differentiation. PC1 and PC2 are involved in the canonical Wnt pathway. After PC1 is cleaved, its CTT binds to β-catenin, and moves into the nucleus to start T cell factor (TCF)-dependent transcription. In the non-canonical Wnt pathway, PC1 plays a role in preserving planar cell polarity, which is crucial for controlled cell division and formation of the kidney tubule. Disruptions in this pathway lead to the enlargement of renal tubules and the development of cysts [22]. 

Tuberous sclerosis complex (TSC) results from a genetic mutation in either the *TSC1* or *TSC2* gene, affecting the production of hamartin or tuberin proteins. This disruption can lead to skin lesions, seizures, and tumors [25]. Tuberous sclerosis complex (TSC) and ADPKD may coexist because the *PKD1* gene is located next to *TSC2,* a gene associated with TSC on chromosome 16 [22]. Normally, polycystin-1 and the *TSC1/TSC2* tumor suppressor genes work together to antagonize mTOR activity, which helps control cell growth by preventing the G1-replication phase of the cell cycle, ultimately leading to apoptosis [25]. This collaboration is mediated via the stabilization of *TSC1-TSC2* complex by PC1 by two different methods. PC1 prevents ERK-dependent phosphorylation of TSC2 which allows it to stop Akt-dependent phosphorylation of TSC2 by attaching to TSC2 at the cell membrane [26]. These interactions allow for the inhibition of the mTOR signaling pathway. 

A dysfunction in the process of tuberin protein transportation of PC1 to the cell membrane may lead to formation of cysts. Therefore, the deletion of *TSC2-PKD1* can lead to ADPKD [25]. The severity of the disease could be attributed to how the genes are expressed, which is based on the influence of microRNAs. In TSC patients with ADPKD, the formation of cysts occurs through a two-hit tumor suppressor process in the cells that make up the cyst wall. In a study conducted by Sampson et al., individuals with deletions in both *TSC1* and *PKD2* may have more severe symptoms, presenting with bigger cystic kidneys and advanced signs of ADPKD when diagnosed [25,27]. 

#### 5.1.3. Mechanisms of Pathogenesis

Individuals with ADPKD affected by PKD1 mutations present with a more severe form of the disease compared to ADPKD-affected individuals with PKD2 mutations. Furthermore, ADPKD symptoms tend to appear earlier in individuals with PKD1 mutations compared to those with PKD2 mutations [22]. Various kinds of mutations can lead to ADPKD, and the seriousness of the condition depends on the location of the mutation. Recent research indicates that individuals with truncating mutations tend to experience more severe symptoms than those with non-truncating mutations [22]. 

Cysts only develop in kidney tubules and hepatic bile ducts when germline mutations in PKD1 or PKD2 are present. Conversely, in adult tissues, there is loss of function in both copies of the mutated polycystic gene, ultimately leading to faster cyst formation in specific tubular epithelial cells. There are more factors that influence how cysts form, including the timing of PKD1 during developmental stages and the severity of the mutations in PC1 [22].

ADPKD is identified by the development of multiple fluid-filled cysts in the kidneys. These cysts enlarge through cellular proliferation, leading to the expansion of renal tubules and eventual kidney failure. Through studies with mice, one model for the development of cysts in ADPKD suggests that kidney-specific PKD1 or PKD2 mutations result in the loss of oriented cell division. This model proposes that a defect in planar cell polarity determines the extent of dilation of cysts, but does not initiate their formation [22]. Other studies indicate that the start of cyst formation relies on cAMP-stimulated Cl- transport in the cells lining the cysts. Elevated levels of cAMP can activate Cl-transport, leading to Cl-driven fluid secretion, and therefore dilating renal tubules. Polycystin proteins play a role in controlling cAMP signaling by influencing the location and activity of chloride channels [22]. Furthermore, ADPKD is widely regarded as a ciliopathy due to the abnormal primary cilia function and its role in cyst formation. Experiments using mouse models with either PKD1 or PKD2 inactivation have revealed that the absence of cilia hinders cyst growth throughout all segments of the nephron. These findings suggest a connection between polycystin and cilia in the pathogenesis of ADPKD and cyst formation [28].

#### 5.1.4. Clinical Presentation/Diagnosis

ADPKD typically becomes clinically evident in adulthood, but cysts and enlarged kidneys can occur at any age including in fetuses or neonates. The common initial symptoms are hypertension, starting around the age of 30–34, accompanied by urinary issues like infections and kidney stones [20]. In children, ADPKD may manifest with macrohematuria, hypertension, UTIs, polyuria, and polydipsia [29]. Additionally, rare complications include cerebral aneurysms at the middle cerebral artery, anterior communicating artery, and internal carotid artery [30]. 

Renal ultrasonography is the primary method for diagnosing ADPKD, which reveals multiple cysts and hyperechoic tissue [1]. The occurrence of a single simple cyst in a child with significant family history is indicative of ADPKD due to the rarity of renal cysts in the pediatric population. For adults, the “Ravine criteria” has been a diagnostic tool for ADPKD, taking into consideration the age and cyst count of the patient [31].

De novo mutations are suspected in individuals with ADPKD and a negative family history. For instances of ADPKD cases due to de novo mutations or situations like kidney transplantation, genetic analysis may be performed for diagnosis [1]. Tolvaptan’s efficacy was evidenced by lower total kidney volume (TKV) elevation in the Tolvaptan Efficacy and Safety in Management of Autosomal Dominant Polycystic Kidney Disease and Its Outcomes trial (TEMPO 3:4).

#### 5.1.5. Management

Tolvaptan is an approved treatment for ADPKD and acts via V2 receptor antagonism, effectively delaying TKV growth and therefore slowing disease progression and mitigating pain. However, hepatoxicity can present as a side effect, and is observed in 11% of patients [32]. Furthermore, additional reported side effects include dehydration, increased urination, and elevated uric acid [32]. Tolvaptan’s efficacy was evidenced by lower TKV elevation in the Tolvaptan Efficacy and Safety in Management of Autosomal Dominant Polycystic Kidney Disease and Its Outcomes trial (TEMPO 3:4) [33]. However, tolvaptan use for the ADPKD pediatric population lacks FDA approval due to developmental effects. Therefore, symptom-focused management is the preferred treatment for children affected by ADPKD [33]. Starting tolvaptan early in adults with ADPKD is recommended to slow the progression of renal failure. [1]. An additional therapy is octreotide, a somatostatin agonist that inhibits cAMP. Octreotide has demonstrated favorable effects on liver disease in adults with ADPKD [20].

Therapeutic approaches for ADPKD focus on addressing cardiovascular symptoms, suggested to occur due to abnormalities in RAAS. ACE inhibitors and ARBs are considered primary treatments for hypertension and proteinuria in pediatric cases [1]. Lifestyle modifications including weight management and a customized diet are recommended to support treatment efforts. If conservative measures are ineffective, surgical options such as cyst aspiration, kidney removal, and decortication can be considered [32].

### 5.2. Autosomal Recessive Polycystic Kidney Disease

#### 5.2.1. Genetics

Autosomal recessive polycystic kidney disease (ARPKD) is a rare hereditary cystic kidney disease with a prevalence of 1 in 20,000 births, which is less common than ADPKD. A comparison of these two diseases is highlighted in Table 1. ARPKD typically appears in neonates or childhood [34]. ARPKD results from mutations in *PKHD1* and *DZIP1L*, which, respectively, produce fibrocystin (FPC) and DZIP1L, both located in the cilia [34]. 

#### 5.2.2. PKHD1 Gene and Fibrocystin Product

*PKHD1* is an extensive gene located on chromosome 6p12 with 86 exons, forming complex alternative splice variants and a large mRNA product. *PKHD1* is expressed in many areas including renal, pancreatic, and hepatic tissue. The encoded protein, fibrocystin (FPC), consists of 4074 amino acids and is composed of two components: a large extracellular domain containing the N-terminal region and a small intracellular domain containing the C-terminal region [34]. The extracellular domain comprises twelve TIG/IPT domains that serve as cell surface receptors, whereas the intracellular domain includes sites for protein kinase A (PKA) phosphorylation [35]. FPC is positioned on the ciliary membrane and plays a role in guiding ciliary targeting. *PKHD1* structural features and the observed histological abnormalities in ARPKD individuals indicate that fibrocystin plays a role in governing cell proliferation and adhesion. During renal development, FPC is initially located at the apical aspect of nephronic precursors, but later concentrates in the basal body in the early stages of ciliogenesis [36]. This specific arrangement over time implies that FPC may be implicated in microtubule organization and the detection of mechanical or chemical stimuli, which are essential functions of primary cilia [36].

Recent evidence points to various FPC isoproteins that might be released in exosomes and undergo processing after translation. However, it is unclear how many alternative *PKHD1* transcripts actually translate into functional proteins. To grasp the role of individual isoforms in maintaining renal and hepatobiliary function, we need a better understanding of FPC in ARPKD pathophysiology [36].

Nearly 750 mutations have been identified in the *PKHD1* gene, with about half of them being missense mutations. The most common mutation, found in more than 20% of cases, is a missense mutation in exon 3 (c.107C>T; p.Thr36Met) and has a severe presentation [35]. Due to the high occurrence of compound heterozygotes and multiple alleles, deciphering consistent genotype–phenotype correlations has been difficult. Therefore, instead of pinpointing specific mutation sites, correlations often emphasize the type of mutation [36]. The main types of variants of *PKHD1* are the truncating, missense, and intronic/splice mutation [35]. Having two truncating mutations is linked to the most severe outcomes. On the other hand, a less severe phenotype is associated with having two missense mutations or inheriting a missense mutation alongside a truncation [34]. The severe presentation of patients with two severe truncating alleles is likely due to the loss of function of FPC. Normal function of FPC demands a specific quantity of the FPC protein, and this cannot be compensated for by alternative isoforms [36].

#### 5.2.3. DZIP1L Gene and DZIP1L Product

Analyzing the *DZIP1L* gene is more straightforward than analyzing *PKHD1* due to its smaller size (of only 16 exons). *DZIP1L* produces a 767-amino acid protein, DAZ (deleted in azoospermia) interacting protein 1-like, which is found in the ciliary transition zone. DAZIP1L includes a zinc finger protein with multiple coiled-coil domains and one C2H2-type zinc finger domain located by its N-terminus [35]. Similar to other PKD proteins, it is located at centrioles and the distal end of basal bodies [36]. The role of DZIP1L in primary cilium formation and possible function in the trafficking of polycystin suggests a connection to ARPKD pathogenesis [35]. Due to the smaller size of *DZIP1L*, mutations causing ARPKD are much less common than compared to *PKHD1* mutations. Additionally, *DZIP1L* mutations are more likely in the region containing the N-terminus [36]. Dysfunctional DZIP1L proteins may prevent the trafficking of PC1 and PC2 to the ciliary axoneme, causing the buildup of PC1 and PC2 in the ciliary basal body/transitional zone [34]. Although *DZIP1L* mutations are not a frequent cause of ARPKD, NGS diagnostic multigene panels should still focus on this gene due to its potential to interact with other PKD or ciliopathy loci and to enhance our understanding of ARPKD [35].

#### 5.2.4. Pathophysiology

The specific processes underlying cyst formation in ARPKD are still not fully understood. However, recent studies using animal models have revealed insights into the mechanisms of cyst development and progression, including alterations in the cAMP pathway via PKA kinase, fluid secretion, and in the extracellular matrix (ECM) [35].

#### 5.2.5. EGFR Axis Expression and Epithelial Secretion

ARPKD has been linked to elevations in the expression of epidermal growth factor (EGF), epidermal growth factor receptor (EGFR), or transforming growth factor-α (TGF-α) levels [35]. The overexpression of these proteins on renal cystic epithelium is linked to ARPKD in rodent models, leading to the formation of renal and hepatic cysts. Studies have demonstrated that reducing EGF tyrosine kinase activity led to improved renal function and decreased cyst formation [35].

Epithelial secretion is another contributing factor for cyst formation. According to Ilatovskaya et al., the role of sodium in cyst formation is unclear, and it is uncertain whether cystic cells enhance or reduce sodium secretion [37]. However, research suggests that sodium uptake plays a role in the development of high blood pressure in ARPKD. This process is facilitated by the epithelial sodium channel (ENaC), which controls sodium reabsorption in the collecting ducts. A study observed increased ENaC activity and expression in ARPKD, ultimately contributing to higher sodium reabsorption and hypertension [38]. A study demonstrated that the lack of CFTR did not impede the advancement of cysts in the kidney and liver, indicating that processes involving Cl- and fluid secretion contrast between ADPKD and ARPKD [35].

#### 5.2.6. cAMP Pathway

Certain studies have shown that cAMP triggers the growth of renal epithelial cells in both ARPKD and ADPKD [35]. The cAMP mechanism activates various pathways (MEK, ERK, and B-Raf), while also increasing the intracellular pathways (MAPK and AKT/mTOR), thus resulting in a reduction in intracellular calcium. Studies have shown that inhibiting intracellular calcium led to increased cell proliferation in cultured ARPKD cells [35]. The imbalance in calcium levels in PKD leads to an increase in vasopressin V2 receptor, triggering the cAMP/PKA cascade. The effectiveness of V2 receptor antagonists was shown to lower renal cAMP levels and mitigate the adverse effects of cystic renal disease [35]. These findings demonstrated the potential for future treatment strategies to focus on interventions targeting calcium levels.

#### 5.2.7. Cilia

The positioning of the ARPKD proteins, specifically the zinc finger protein DZIP1L and FPC, are located within the cilia. Cilia are elongated microtubular structures on the surface of cells. ARPKD is a condition arising from improper cilia function, falling under the category of ciliopathies [35]. Calcium is an important component of the cilia process and is transported via PC2, which is a member of the transient receptor channel (TRP) [39]. Fibrocystin typically plays a role in controlling PC2 and therefore the activity of cilia. The absence of *PKHD1* has been demonstrated to change the activity of PC2. Despite these findings, current research has not established the importance of the PC2 binding domain in FPC for fibrocystin function [40]. 

Through studies in various animals, the DZIP1L protein has been observed to reside within centrioles and the transition zone of primary cilia [35]. DZIP1L engages with septin 2 (SEPT2) protein, contributing to the maintenance of diffusion at the transition zone of cilia. Within mutant DZIP1L cells, the movement of PC1 and PC2 from the basal body to the ciliary axoneme was changed; instead of their usual distribution in the ciliary axoneme, both proteins were confined to the basal body [35]. However, the absence of *DZIP1L* did not change the placement or expression of FPC. These results indicate *DZIP1L’s* involvement in the transportation of polycystins and demonstrate the linkage of ARPKD with ADPKD [35].

#### 5.2.8. Clinical Presentation

ARPKD typically presents earlier and is more detrimental compared to ADPKD, commonly featuring bilateral renal cysts, enlarged and echogenic kidneys, and hepatic fibrosis potentially causing portal hypertension [36]. The most pronounced phenotypes are observed in early diagnoses; however this condition has variable onset, as it may manifest during perinatal, neonatal, childhood, or young adulthood stages. Oligohydramnios is associated with ARPKD and can result in a “Potter sequence”, marked by pulmonary hypoplasia, limb and facial deformities, and occasional fetal death. Nearly 50% of newborns with pulmonary hypoplasia due to ARPKD experience respiratory failure, and nearly 30–40% of pediatric ARPKD patients do not survive beyond the first year [34,36].

Addressing arterial hypertension within the initial year is crucial in ARPKD, and requires pharmacological intervention to safeguard the kidneys [35]. Early concerns also involve UTIs, gross hematuria, renal osteopathy, and gross hematuria. Ultrasound detection reveals enlarged kidneys due to distal nephron dilatations within the collecting duct. Over time, the structure of the kidney resembles ADPKD, and progression to ESRD occurs in nearly half of individuals [35].

ARPKD not only affects the kidneys, but also entails cystic liver involvement, reflected in the mutated gene’s name, “polycystic kidney and hepatic disease 1” (*PKHD1).* The hepatic manifestation clinically presents as congenital hepatic fibrosis (CHF) and involves bile duct dilatations, which contribute to portal hypertension, cholangitis, an enlarged spleen, esophageal varices, and thrombocytopenia [36].

#### 5.2.9. Diagnosis

Early manifestation of ARPKD is often diagnosed prenatally through renal ultrasonography in the second or third trimester, revealing echogenic renal enlargement with medullary hyperechogenicity [35]. Prenatal diagnosis is selectively offered based on an increased risk of ARPKD, because it requires invasive procedures like amniocentesis or chorionic villus sampling. The use of NGS has proven effective in genetic testing for ARPKD due to its ability to navigate allelic variations and handle the large genomic size [36].

ARPKD can arise from various recessive and dominant genes, and may mimic the effects of *DZIP1L*, *PKD1* and *PKD2*, *TSC2*, *HNF1β*, and nephronophthisis (NPHP) gene mutations [35]. Notably, *DZIP1L* mutations typically present with a milder phenotype and lower risk of perinatal death. Although ARPKD and ADPKD share many clinical features, distinguishing factors include cardiovascular events being more common in ADPKD mutations, while hepatobiliary abnormalities are more often associated with ARPKD mutations. Mutations in *TSC1* and *TSC2* give rise to tuberous sclerosis and are associated with death due to renal complications. Polycystic kidney disease emerges from deletions in adjacent genes *PKD1* and *TSC2* on chromosome 16p [35]. Furthermore, mutations in *HNF1β/TCF2* may lead to Potter’s sequence and renal enlargement with cysts. Nephronophthisis (NPHP) is an autosomal recessive disorder characterized by renal fibrosis and the presence of tubulointerstitial cysts. Unlike ARPKD, kidneys affected by NPHP do not undergo enlargement. Mutations resembling ARPKD are often linked to ciliopathies, including Bardet–Biedl, Joubert, and Meckel syndrome [35].

#### 5.2.10. Management

Currently, clinical trials are exploring the use of Tolvaptan, a selective antagonist of the V2 receptor, for the treatment of ARPKD. Another option being studied is Tesevatinib (TSV), which acts as an antagonist for the EFGR axis and VEGF receptors [35].

### 5.3. Nephronophthisis

Infantile nephronophthisis (NPHP), or type 2 NPHP, is an autosomal recessive disorder characterized by a severe phenotype leading to kidney failure before the age of four [11]. The incidence of NPHP within the United States is estimated to be 1 in 1,000,000 [41]. NPHP is attributed to recessive mutations in genes such as *NPHP2/INVS* and *NPHP3*, while mutations in NPHP9/NEK8, *ZNF423/NPHP14*, and *ANKS6/NPHP16* are less frequently observed [1]. The majority of genes that lead to NPHP are involved in encoding the primary cilium. The ciliary signal transduction process if pivotal for kidney development, and dysfunction within this pathway is linked to cyst formation and renal dysfunction [42]. Additionally, a source of cAMP is the vasopressin receptor type 2 (V2R) which is encoded by AVPR2. VR2 is a G protein-coupled receptor that plays a crucial role in renal physiology. Impairment of this pathway can explain the urine concentration defect that occurs in NPHP, which resembles nephrogenic diabetes insipidus that also results from mutations in AVPR2 [42]. Kidney enlargement and reduced amniotic fluid levels may be identified during pregnancy accompanied by elevated blood pressure, ventricular septal defects, and situs inversus. NPHP in infants may resemble polycystic kidney disease because of interstitial fibrosis and tubular wasting. The infantile variant is characterized by enlarged renal cysts and lacks histological anomalies in the tubular basement membrane, distinguishing it from other forms of the disease [1]. The most common form is juvenile NPHP, which presents with kidney failure around age 13 [41]. Diagnosis of NPHP is typically made with kidney ultrasonography, which reveals regular kidney size, increased echogenicity, and poor cortico-medullary differentiation. Neonatal NPHP may display enlarged kidneys bilaterally [41].

Currently, there is no established treatment for NPHP, and the main approach involves supportive care or kidney transplantation. Ongoing research is exploring the potential use of vasopressin V2 receptor antagonists, like tolvaptan, as a potential treatment for renal cysts associated with the *NPHP3* gene. Additionally, inhibitors targeting mTOR are also being explored for their applicability in NPHP [1].

### 5.4. Bardet–Biedl Syndrome

Bardet–Biedl syndrome (BBS) is a ciliopathy impacting various systems with an incidence of 1:100,000 in North America and Europe [43]. Although there are some instances of potential oligogenic inheritance, the predominant inheritance pattern follows an autosomal recessive pattern. Clinically diagnosed BBS cases are predominantly associated with over 20 identified genes, ranging from *BBS1* to *BBS21* and encompassing more than 95% of cases. Specifically, 23.2% of BBS cases are attributed to the *BBS1* gene, while *BBS10* accounts for nearly 20% of cases [1]. Eight BBS proteins, namely BBS1, 2, 4, 5, 7, 8, 9, and 18, form the BBSome, which is a regulator of the composition of ciliary membrane proteins. The involvement of BBS proteins with the primary cilium demonstrates its potential role in the pathogenesis of the renal dysfunctions associated with BBS [44]. BBS may manifest with kidney abnormalities such as horseshoe kidneys, fetal lobulation, kidney dysgenesis, and cysts. To achieve a definitive diagnosis, genetic testing is necessary due to the resemblance of PKD or NPHP on ultrasound [1].

A clinical diagnosis of BBS relies on various symptoms and manifestations. Key clinical features include hypogonadism, renal parenchymal abnormalities, polydactyly, rod-cone dystrophy, and truncal obesity [44]. BBS has variable phenotypes; however, truncal obesity and retinal dystrophy are the most common and consistent presentations. Additional complications of BBS encompass diabetes mellitus, Hirschsprung disease, dental abnormalities, and hepatic fibrosis. BBS on average is typically diagnosed around the age of nine years [44].

Currently, there is no specific treatment for protocol for BBS; however, management focuses on blood pressure regulation, renal ultrasound imaging, and assessing endocrine hormone levels Recently, based on a case series, early educational interventions to tackle retinal dystrophy and the progression towards blindness have been suggested, with measures such as Braille training, large-print reading, and computing skills [43].

### 5.5. Meckel–Gruber Syndrome 

Meckel–Gruber Syndrome (MKS) is an autosomal recessive disorder resulting from mutations impacting the structure and function of the primary cilium. The global incidence of MKS ranges between 1 in 13,250 to 1 in 140,000 live births [28]. Mutations in 14 genes have been identified as contributors to MKS, with Meckelin mutations being the most prevalent and accounting for 16% of MKS cases [28]. Meckelin plays a crucial role in centrosome migration and subsequent development of primary cilia. Meckelin is typically found in the primary cilia, basal body, and plasma membrane [28]. Although MKS is very rare, it is recognized as the most severe type of ciliopathy, characterized by malformations in the posterior fossa, cystic enlargement of the kidneys, polydactyly, and irregular liver development [28]. The predominant hallmark of MKS is cystic dysplasia resulting in significant abdominal distension due to the large kidneys. Imaging of patients with MKS can demonstrate oligohydramnios. NGS and chorionic villus sampling are employed for a definitive diagnosis of MSK [1]. Due to the innovations in ultrasound technology and radiology, diagnosis of MKS is typically made in the first trimester, with detection of the classic manifestations mentioned above. Additionally, the diagnosis can be supported through the detection of increased maternal serum α-fetoprotein levels [45].

### 5.6. Autosomal Dominant Tubulointerstitial Kidney Disease

#### 5.6.1. Genetics and Pathophysiology

Autosomal dominant tubulointerstitial kidney disease (ADTKD) is a genetic disorder that encompasses tubular damage, interstitial fibrosis, and progression to ESRD without glomerular lesions. Recent developments in genetic testing indicate that ADTKD contributes to almost 5% of monogenetic disorders associated with CKD [46]. Currently, ADTKD is categorized into six distinct genetic inheritance anomalies: ADTKD-*UMOD*, ADTKD-*MUC1*, ADTKD- *REN*, ADTKD-*HNF1B*, ADTKD-*SEC61A1*, and ADTKD-*DNAJB11*. These are shown in Table 2. Due to its rarity and diagnostic challenges, additional classifications might emerge in the future. ADTKD-*UMOD* stands out as the most prevalent variant, constituting 0.3% of genetically linked renal diseases worldwide. ADTKD-*MUC1* represents the second most frequently encountered form, with a prevalence of 21% [47].

#### 5.6.2. ADTKD-UMOD

In an Austrian survey, the most common subtype of ADTKD was ADTKD-*UMOD*, demonstrating a prevalence of 1.7 cases per million in the population [48].

Additionally, findings from European and American registries indicated a 37% prevalence of ADTKD-*UMOD* among 726 individuals diagnosed with ADTKD [47].

##### Pathophysiology

Uromodulin (UMOD) is a glycoprotein found exclusively in the kidney on luminal epithelial cells of the thick ascending loop (TAL) and early distal convoluted tubule (DCT). It plays a crucial role in maintaining water balance within the kidneys through its regulation of water permeability. UMOD forms gel-like structures that influence water permeability and control the activity of the renal outer medullary potassium channel and the sodium–potassium–chloride cotransporter. UMOD has a protective effect against the formation of kidney stones due to its inhibition of crystallization of calcium oxalate crystallization and its ability to defend against bacteria [49]. Additionally, recent evidence suggests that UMOD regulates the migration of neutrophils in cases of acute kidney injury [50].

ADTKD-*UMOD* is considered a storage disease arising secondary to the trapping of misfolded uromodulin in the endoplasmic reticulum (ER). This results in the formation of substantial aggregates and the enlargement of ER stacks observed in renal biopsy [51]. Numerous studies using in vitro and in vivo models have demonstrated that the main impact of UMOD pathogenic variants is the retention of mutant uromodulin in the ER. Several models highlighted the triggering of ER stress and the unfolded protein response (UPR), which are cellular mechanisms to eliminate ER stress [49]. The persistent presence of misfolded proteins causing chronic ER stress in the cells of the TAL is responsible for the disruption in mitochondria and inhibition of autophagy. In mouse models representing ADTKD-*UMOD*, the accumulation of mutant uromodulin in the ER demonstrated deterioration of the kidneys. This deterioration mirrored the clinical characteristics of ADTKD-*UMOD* such as tubulointerstitial fibrosis, defects in urinary concentration, dilation of tubules, and eventual renal failure [52]. UMOD-knockout mice did not exhibit these clinical characteristics, demonstrating that the pathophysiology of uromodulin variants involves a toxic gain-of-function mutation [53]. These variants displayed evidence of UMOD excretion onto the plasma membrane and subsequent disruption of TAL membrane integrity. Consequently, this causes a shortage in urine concentration and sodium reabsorption, leading to hypovolemia. Hypovolemia can result in increased uric acid reabsorption in the PCT, leading to hyperuricemia [49].

The main impact of UMOD pathogenic variants such as ER retention and stress is well studied; however, the downstream effects are largely unknown. The presence of inflammatory markers in the early stages of ADTKD mouse models suggest that inflammation plays a role in the onset of disease, as it occurred before the initiation of kidney damage. 

Considerable variability exists in the age of onset and progression, making it challenging to establish a correlation between genotype and phenotype. Nevertheless, certain risk factors linked to an earlier onset of ESRD have been identified, such as male sex and extent of the trafficking defect in variant UMOD [49].

##### Clinical Presentation

ADTKD-*UMOD* is characterized by a rise in serum creatinine levels, a negative urinalysis with mild proteinuria, and bland urinary sediment with occasional histological similarities to focal segmental glomerular sclerosis [49]. In the TAL, electron microscopy reveals uromodulin accumulation as substantial intracellular deposits. Kidney size diminishes over time, and is visualized with ultrasonography. The onset of ESRD typically occurs around the age of 54, with a less favorable prognosis for men [47]. Preceding the development of CKD, elevated blood uric acid levels are common (70–80%) and often lead to gout in 20–74% of patients [49].

##### Diagnosis

For a conclusive diagnosis, genetic testing using NGS is employed when a heterozygous pathogenic variant in the *UMOD* gene is detected. Over 130 pathogenic variants have been identified, and predominantly involve missense mutations in exons 3 and 4, which results in the substitution of cysteine residues and misfolding of the uromodulin protein [49].

##### Management

Management for ADTKD-*UMOD* is not well established, but typically focuses on addressing CKD and gout. Allopurinol is prescribed for gout-related issues, while CKD is managed through controlling high blood pressure and addressing anemia, elevated serum phosphate, and ESRD. Renal transplantation demonstrates effectiveness as it prevents the recurrence of ADTKD-*UMOD* [49]. Another treatment being explored is TNFR:Fc, an inhibitor targeting TNF- α, which slows the progression of ADTKD-*UMOD* in mice using its anti-inflammatory properties [54].

#### 5.6.3. ADTKD-MUC1

ADTKD-*MUC1*, also known as medullary cystic kidney disease (MCKD), relies on specialized genetic testing that is not frequently utilized and is distinct from NGS, making it difficult to establish prevalence. According to extensive registries in the United States and Europe, ADTKD-*MUC1* emerges as the second most prevalent form of ADTKD, with an estimated prevalence of 21% [49].

##### Pathophysiology

*MUC1* produces mucin1a glycoprotein found on the apical surface of various cells in organs like the lung, intestine, breast, and kidney. Mucin1 serves as a protective barrier and plays a role in cellular signaling. Mucin1 is found on the DCT, TAL, and collecting ducts in renal tissue. The cytoplasm of tubular epithelial cells accumulates abnormal MUC1-frameshift (fs) protein aggregates, which can lead to cell death and the development of tubulointerstitial fibrosis and CKD [49]. Every pathogenic variant in MUC1 results in the formation of the same abnormal protein, which demonstrates that the MUC1-fs protein is important in the development of ADTKD. The absence of clinical symptoms of ADTKD-*MUC1* in mice lacking the *MUC1* gene implies that the disease mutation induces the accumulation of mucin1 and is caused by a gain-of-function mutation [49]. Although the precise pathophysiology of ADTKD-*MUC1* is unknown, there are indications that the accumulations of MUC1-fs in the ER and Golgi apparatus play a role in disease process. These accumulations may activate the unfolded protein response, particularly the transcription factor 6 (ATF6) component [49]. Recently, the compound BRD4780 was found to bind to the TMED9 receptor, facilitating the expulsion of MUC1-fs to the lysosome and its subsequent elimination from cells [55].

##### Clinical Manifestations

ADTKD-*MUC1* has similar clinical features to ADTKD-*UMOD*, like unremarkable urinalysis, elevated serum uric acid causing gout, and CKD. One study revealed that the ADTKD-*MUC1* variant is associated with a more severe renal disease compared to ADTKD-UMOD (58% vs. 44%, respectively, with ESRD) and an earlier onset (36 years vs. 46 years) [47,49]. However, the ADTKD-*MUC1* variant is less commonly linked with gout than the ADTKD-*UMOD* variant (26% vs. 79%) [47,49].

##### Diagnosis

Confirmation of ADTKD-MUC1 diagnosis relies on the detection of a heterozygous *MUC1* pathogenic variant. Genetic testing is challenging due to a large amount of variable-number tandem repeats (VNTR) region on the MUC1 gene. Successful identification of the mutation has been achieved through long-read single-molecule real-time (SMRT) sequencing [49]. Typically, the mutation involves the duplication of cytosine among seven cytosines. The consistent fs mutation in the MUC1 pathogenic variant results in the formation of a truncated protein called MUC1-fs. Immunohistochemistry can be utilized as a diagnostic method to identify MUC1-fs protein in renal biopsy or urinary epithelial cells, offering a viable option for patients lacking the cytosine mutation [49].

##### Management

While there is currently no established therapy for ADTKD-*MUC1*, BRD4780 can possibly be used to clear accumulated MUC1-fs protein from cells. However, additional trials are essential to validate the efficacy of BRD4780, as this evidence is derived from a preclinical study [55].

#### 5.6.4. ADTKD-REN

ADKTD-REN, also referred to as familial juvenile hyperuricemic nephropathy type 2 (FJHN2), is associated with mutations in the *REN* gene, which leads to the synthesis of renin [49]. Renin plays a vital role in the renin–angiotensin–aldosterone system (RAAS) to maintain sodium balance and regulate blood pressure and erythropoiesis. The juxtaglomerular apparatus (JGA) is the site of renin production and its precursor.

##### Pathophysiology

During the synthesis of renin, pre-prorenin undergoes a transformation in the ER in which the signal peptide is removed to form prorenin. The signal peptide plays a role in facilitating the translocation of pre-prorenin to the ER. The prosegment assists in ensuring the correct folding of the protein and is found adjacent to the signal peptide on pre-prorenin. Research has revealed that dominant heterozygous mutations in *REN* result in reduced pro-renin levels, and thus a decrease in renin production [56]. They explained that the unaffected allele enables renin production necessary for proper kidney development but insufficient for a functioning RAAS, resulting in low blood pressure, elevated potassium levels, anemia, and renal tubulointerstitial fibrosis. Signal sequence mutations disrupt the proper insertion of pre-prorenin into the ER [49]. The accumulated mutated mature renin in the ER lacks proper protein folding, which triggers the unfolded protein response and results in cell death, a decline in eGFR, and tubulointerstitial fibrosis. Ultimately, mutations affecting the prosegment lead to the buildup of prorenin and renin in the ER and Golgi apparatus, resulting in diminished secretion of prorenin [56].

##### Clinical Manifestations

ADTKD-REN lacks distinctive laboratory or histologic features except for diminished renin immunostaining in the JGA. It is characterized by reduced renin secretion, consequently leading to decreased aldosterone, elevated serum potassium levels, and mild hypotension. In children, a combination of ADTKD-REN with NSAID use and volume depletion heightens the risk of acute kidney injury. This is due to the inhibition of prostaglandin formation because of NSAID use, which contributes to a constricted afferent arteriole. The constriction of the afferent arteriole would lead to a decreased eGFR in the context of diminished renin and volume [49].

##### Diagnosis

ADTKD-REN diagnosis is based on clinical features such as early onset CKD, hypoproliferative anemia, and later-onset gout. Decreased plasma renin and aldosterone levels, along with increased plasma potassium, are indicative findings of ADTKD-REN. Although urinalysis and renal ultrasonography lack distinctive features, renal biopsy can be used as a diagnostic tool as it may reveal tubular fibrosis. The conclusive diagnosis is established through genetic testing via Sanger and NGS [49].

##### Management

Management of ADTKD-REN encompasses the regulation of blood pressure, anemia, gout, CKD, and potassium imbalance. Fludrocortisone can be used for treatment due to its mineralocorticoid-like effects and ability to stimulate erythropoiesis [49]. The potential role of BRD4780 in future thepaeutic strategies is undergoing further clinical trials [55].

#### 5.6.5. ADTKD-HNF1B

The HNF1B gene encodes hepatocyte nuclear factor 1β and is located in renal, hepatic, and pancreatic tissues. When mutated, it can cause issues such as incomplete pancreas development, genitourinary deformities, gout, and renal issues [49].

##### Pathophysiology

The *HNF1B* gene plays a significant role in various renal processes, impacting aspects like ion movement, nephrogenesis, and the regulation of cystic disease genes such as *GLIS2*, *UMOD*, *PKD2*, and *PKHD1* [49]. The vast involvement of *HNF1B* could explain the diverse clinical manifestations associated with the disease. Although the precise mechanism by which mutated *HNF1B* contributes to renal fibrosis is not entirely known; recent findings suggest that it is involved in the epithelial–mesenchymal transition. This process plays a crucial role in tissue regeneration, but persistent activation is linked to fibrogenesis. Given that half of ADTKD-*HFN1B* patients exhibit heterozygosity for full gene deletion, it can be concluded the phenotype could be attributed to haploinsufficiency [49].

##### Clinical Manifestations

HNF1B-disease exhibits diverse clinical manifestations depending on the age of symptom onset. Prenatal cases may manifest as hyperechogenic kidneys on fetal ultrasonography. In childhood, it can present as HFN1B-nephropathy, which is characterized by renal cystic hypodysplasia, urinary tract malformations, and reduced levels of serum potassium and magnesium. In adulthood, it features increased serum uric acid levels, leading to gout and CKD that may progress to ESRD [49]. ADTKD-*HNF1B* resembles other ADTKD types, but displays variable renal abnormalities such as urinary tract malformations, cysts, and decreased serum magnesium and potassium. Additionally, extrarenal manifestations such as genitourinary abnormalities, MODY5, neurodevelopmental disorders, and an underdeveloped pancreas may be associated with this disease [57].

##### Diagnosis

Diagnosing ADTKD-*HNF1B* is challenging due to its resemblance to other kidney disorders, but extrarenal manifestations and familial variations in phenotypes can provide diagnostic clues. Genetic testing, specifically utilizing conventional sequence analysis and multiplex ligation-dependent probe amplification to identify whole-gene deletion is essential for a definitive diagnosis [49]. Whole-gene deletions are a molecular defect observed in nearly half of patients including cases associated with 17q12 recurrent deletion syndrome. In remaining instances, point pathogenic variants, most specifically missense mutations, can cause ADTKD. De novo pathogenic variants can also account for cases of ADTKD, which can diminish the significance of a positive family history in comparison to other forms of ADTKD [49].

##### Management

Currently, there is limited knowledge regarding the modulation of *HNF1B* gene, leading to difficulties in establishing treatment. Current treatment approaches primarily focus on addressing extrarenal complications, and are supportive for CKD [49]. For individuals experiencing early-onset gout, allopurinol or febuxostat is recommended. Additionally, screening for abnormal liver function and other electrolyte abnormalities is crucial to avert additional complications [58].

#### 5.6.6. ADTKD-SEC61A1

ADTKD-*SEC61A1*, also identified as familial juvenile hyperuricemic nephropathy type 4, (FJHN4), has been observed in a very small number of individuals, with only four cases reported thus far [49].

##### Pathophysiology

The *SEC61A1* gene encodes the α (SEC61A1); β (SEC61B); and γ (SEC61G) subunits constituting SEC61, a translocon-associated protein-conducting channel. This channel plays a crucial role in the transportation of proteins to the ER. In ADTKD-*SEC61A1,* the SEC61 channel undergoes modifications, resulting in the buildup of variant SEC61α in the ER and Golgi apparatus [49]. The accumulation of this variant will result in changes to post-translational modifications and folding processes of proteins such as renin, mucin 1, and uromodulin. A recent investigation revealed that mutations associated with ADTKD- *SEC61A1* cause dysfunction in protein ER translocation, specifically affecting renin [49]. Sicking et al. proposed that the presence of mutant SEC1A1 is associated with reduced polycystin-2 levels, potentially explaining the observed decline in calcium [59]. Bolar et al. explained the potential involvement of SEC61 and its translocon function in renal development, particularly in the pronephros [60]. These disruptions cumulatively can trigger ER stress and subsequent cell deal death and interstitial fibrosis, ultimately making it a potential cause for ADTKD.

##### Clinical Manifestations

Bolar et al. reported clinical presentations in two families affected by ADTKD- SEC61A1 [60]. The first family presented with slowly progressing ADTKD, high uric acid levels, anemia, and several other renal symptoms. Renal imaging revealed shrunken cystic dysplastic kidneys, and renal biopsy showed glomerular sclerosis and tubulointerstitial lesions. Schubert et al. reports that the second family exhibited gout, neutropenia, anemia, and respiratory tract infections due to hypogammaglobulinemia [61].

##### Diagnosis

For a conclusive diagnosis of ADTKD-*SEC61A1,* Sanger next-generation sequencing can be used [49].

##### Management

As of now, there is no established treatment for this condition. Sodium phenylbutyrate has been proposed to enhance renin transport; however, additional trials are needed to determine efficacy [59].

#### 5.6.7. ADTKD-DNAJB11

ADTKD-DNAJB11 is very rare and has been documented in seven families, and featured renal symptoms similar to other ADPKD disorders. These include liver and kidney cysts, renal enlargement, and eventual ESRD [49].

##### Pathophysiology

The *DNAJB11* gene plays an essential role in generating GRP78/BiP, a key participant in organizing and manipulating proteins to provide homeostasis within the ER. Research has established a connection between *DNAJB11* and ADPKD and ADTKD. Experiments involving *DNAJB11* null human renal cortical tubular epithelial cells demonstrated impaired synthesis of the PKD1 protein. Additionally, histology of kidney samples showed irregular intracellular accumulation of uromodulin and mucin1. Collectively, these factors establish a connection to the pathologic process of tubulointerstitial disease and linkage to ADTKD [49].

##### Clinical Manifestations

Histological examination reveals tubulointerstitial fibrosis. Additionally, cases of gout have been documented in affected individuals [62].

##### Diagnosis

A conclusive diagnosis is attainable through either NGS or Sanger sequencing [49].

#### 5.6.8. Genetic Counseling

Given the autosomal dominant nature of ADTKD, there exists a 50% likelihood of inheriting the condition for individuals at risk. Genetic counseling, particularly emphasized for HNF1B mutations due to variable symptoms, is crucial for diagnosis. While conducting genetic testing using multiplex ligation-dependent probe amplification for the HNF1B gene and NGS for other genes, it is vital to note that a negative result does not rule out ADTKD [49].

## Figures and Tables

**Table 1:** Comparison of ADPKD and ARPKD.

Disease Aspect	ADPKD	ARPKD
Mutated gene > encoded product	PKD1 > polycystin-1 (PC1) PKD2 > polycystin-2 (PC2)	PKHD1 > fibrocystin (FPC)
Common age of onset	Adult	Neonate and childhood
Inheritance	Autosomal dominant	Autosomal recessive
Family history of PKD	Commonly present	Commonly absent
Macroscopic cysts	Present	Few or none
Other clinical symptoms	Hypertension, urinary concentrating defect, hematuria, cysts outside of kidney Less common symptoms: urolithiasis, flank pain, mitral valve prolapse, cerebral/aortic aneurysm	Hypertension, urinary concentrating defect, hepatosplenomegaly Less common symptoms: Portal hypertension
Risk of end-stage renal disease (ESRD)	50% lifetime risk	>60% by adulthood

**Table 2:** Comparison of different genetic disorders in ADTKD.

Genetic Disorder	Unique Clinical Manifestations	Diagnosis	Pathophysiology	Treatment	Other Notes
UMOD	Accumulation of intracellular deposits within the ER in the TAL. Gout and ESRD.	Genetic testing	Uromodulin accumulation	Management of Gout and CKD. Renal transplantation to prevent recurrence. BRD4780 TNFR:Fc.	Most common ADTKD
MUC1	Resembling ADTKD-*UMOD,* but exhibiting increased prevalence of ESRD at earlier age Gout is less common	Genetic testing for the heterozygous *MUC1* variant	Buildup of MUC1-fs Typically 7 cytosine insertion stretch.	BRD4780	Second most common ADTKD
REN	Childhood onset vs. adult onset. Diminished renin immunostaining in the JGA. Reduced erythropoietin, elevated potassium levels	Genetic testing CKD, anemia, and gout manifesting n early childhood	Reduced renin and aldosterone levels.	Fludrocortisone and erythropoiesis-stimulating agents.	
HNF1β	Variable renal and extrarenal manifestations: congenital kidney and urinary tract anomalies, pancreatic irregularities	Genetic testing, reduced magnesium and potassium, gout	Disrupted control of gene transcription for nephrogenesis, ion transport, cystic disease genes	Treat extrarenal anomalies	
DNAJB11	Anemia, low neutrophil levels, reoccurring respiratory tract infections	Genetic testing	Missense mutation, SEC61-channelopathy within ER	Sodium phenylbutyrate	Very rare
SEC61A1	Bilateral small renal cysts, liver cysts	Genetic testing	Disruption of GRP78/BiP cofactor. Possibly PKD1 protein	n/a	Very rare

## Data Availability

No new data were created or analyzed in this study. Data sharing is not applicable to this article.
